# Synthesis of Nickel *In Situ* Modified SAPO-11 Molecular Sieves and Hydroisomerization Performance of Their NiWS Supported Catalysts

**DOI:** 10.3389/fchem.2021.765573

**Published:** 2021-11-22

**Authors:** Xiaojun Dai, Yan Cheng, Meng Si, Qiang Wei, Luyuan Zhao, Xiaohan Wang, Wenbin Huang, Haoran Liu, Yasong Zhou

**Affiliations:** State Key Laboratory of Heavy Oil Processing, China University of Petroleum, Beijing, China

**Keywords:** *in situ* modification, SAPO-11, active phase, catalyst, hydroisomerization

## Abstract

SAPO-11 molecular sieves were modified with different Ni contents by the *in situ* modification method. The Ni-modified SAPO-11 molecular sieves were used as the supports to prepare the corresponding NiW-supported catalysts for the hydroisomerization of n-hexadecane. The Ni-modified SAPO-11 and the corresponding NiW-supported catalysts were characterized by X-ray diffraction, scanning electron microscopy, N_2_ adsorption–desorption, NH_3_-temperature-programmed desorption, pyridine adsorbed infrared, high-resolution transmission electron microscopy, and X-ray photoelectron spectroscopy. The results showed that Ni *in situ* modification preserved the crystal structure of SAPO-11; increased the BET specific surface area, mesopore volume, and medium and strong Brønsted acid amount of SAPO-11; and increased the stacking number of the active phase of the catalysts. 3Ni-SAPO-11 possessed the largest BET specific surface area, mesopore volume, and medium and strong Brønsted acid amount. NiW/3Ni-SAPO-11 possessed the highest dispersion of the active phase and the highest sulfidation degree of the active metals. The results of the hydroisomerization of n-hexadecane showed that Ni *in situ* modification improved the catalytic activity and selectivity of the catalysts for the hydroisomerization of n-hexadecane to varying degrees. Especially, NiW/3Ni-SAPO-11 had the highest catalytic activity and isomer selectivity, and the maximum yield of isomeric hexadecane could reach 71.18%.

## Introduction

In order to meet the utilization standards of oil products in cold regions, diesel oil is required to have a low freezing point, and lubricant base oil has good low-temperature fluidity. The existence of long-chain n-alkanes is considered to be the direct cause of the high freezing point of diesel oil and the poor low-temperature fluidity of lubricant base oil (Regali et al., 2014; Bai et al., 2019). The best way to solve this problem is to hydroisomerize long-chain n-alkanes in oil products to form branched isomers (Lin et al., 2002; Wei et al., 2017). The catalyst in the process of alkane hydroisomerization is a bifunctional catalyst, which is composed of the acid support providing acid centers for the isomerization of the olefin intermediate skeleton and the active metal providing metal centers for dehydrogenation/hydrogenation activity (Chen et al., 2019; L.; Yang et al., 2019).

Silicoaluminophosphate-11 (SAPO-11) is a microporous silicoaluminophosphate molecular sieve made up of three types of tetrahedral units (SiO_2_, AlO_2_^−^, and PO_2_^+^ tetrahedrons) arranged alternately and is a kind of weakly acidic support with an AEL structure and a one-dimensional pore channel, which is considered as the ideal support for the catalyst for the hydroisomerization of alkanes due to its unique pore structure and mild acidity and has been widely used in the hydroisomerization process of the petrochemical industry and achieved good economic benefits (Li et al., 2013; Song et al., 2016). However, how to improve the physical and chemical properties of SAPO-11 and make it have better catalytic performance is still a focus worthy of study by researchers. It is believed that the medium and strong Brønsted acid sites of SAPO-11 are the active sites of the isomerization of the alkane skeleton (Huang, 2003; Zhang et al., 2018). Therefore, increasing the medium and strong Brønsted (B) acid content of SAPO-11 is bound to improve the catalytic performance of SAPO-11. At present, noble metals (Pt, Pd) are the most widely used metal components of bifunctional catalysts for the isomerization of alkane, which are highly sensitive to the sulfur-containing compounds in raw materials during the catalytic reaction, and can easily lead to poisoning and deactivation of catalysts (Yang et al., 2017; Lyu et al., 2019; Lyu et al., 2020). Meanwhile, the high cost of noble metals also increases the cost of preparation of these catalysts, which limits their industrial applications (Xiao et al., 2021). Some researchers have used Ni instead of noble metals to prepare catalysts for hydrocarbon hydroisomerization (Yuan et al., 2020). Although the preparation cost of catalysts has been greatly reduced, Ni-supported catalysts have weaker dehydrogenation/hydrogenation activity and stronger hydrogenolysis activity than noble metal-supported catalysts, which greatly reduces the catalytic activity of catalysts and the selectivity of hydroisomerization. Yuan et al. prepared a SAPO-11-supported Ni catalyst for the hydroisomerization of n-heptane. The results showed that when the conversion of n-heptane was 29%, the selectivity of i-heptane was only 34%, while when the conversion of n-heptane was 45%, the selectivity of i-heptane was only 49% (Yuan et al., 2020). Finding metal components with low cost and high dehydrogenation/hydrogenation activity is the research focus of developing a new SAPO-11 molecular sieve-based hydroisomerization catalyst. It is reported that NiWS and NiMoS active phases have excellent dehydrogenation/hydrogenation performance and have been widely used in hydrodesulfurization catalysts, hydrodenitrogenation catalysts, and hydrocracking catalysts (Díaz de León et al., 2017; Woolfolk et al., 2017; Zhou et al., 2017; Cui et al., 2019). However, there are few reports about the application of NiWS and NiMoS active phases in the SAPO-11 molecular sieve-based hydrocarbon hydroisomerization catalyst. The properties of the active phase, such as slab length, stacking number, and dispersion, are closely related to the catalytic performance of the catalyst.

In this article, SAPO-11 molecular sieves were modified with different percentages of Ni content by the *in situ* modification method, and NiW supported catalysts were prepared for the hydroisomerization of n-hexadecane with the Ni *in situ* modified SAPO-11 molecular sieves as supports. The effects of Ni *in situ* modification on the properties of SAPO-11 and the corresponding catalysts were investigated. The effects of Ni *in situ* modification on the properties of the active phase of SAPO-11-supported NiW catalysts and the effects of the properties of the NiWS active phase on the hydroisomerization performance of the catalysts were investigated for the first time.

## Experimental

### Materials

Phosphoric acid (H_3_PO_4_, 85wt%; Aladdin), pseudo-boehmite (Al_2_O_3_, 70wt%; Macklin), diisopropylamine (DIPA, 99wt%; Aladdin), di-n-propylamine (DPA, 99wt%; Aladdin), acid silica sol (SiO_2_, 30wt%; Dezhou Jinghuo technology Glass Co., Ltd.), dodecyltrimethylammonium bromide (DTAB, 99wt%; Aladdin), nickel nitrate hexahydrate [Ni (NO_3_)_2_·6H_2_O, 98wt%; Aladdin], ammonium metatungstate hydrate ((NH_4_)_6_ H_2_W_12_O_40_·xH_2_O, 99.5wt%; Macklin), hydrogen (H_2_ ≥ 99.999%; Beijing Haipu Gas Co., Ltd.), and deionized water were the materials used.

### Synthesis of *In Situ* Ni-Modified SAPO-11 Molecular Sieves

SAPO-11 molecular sieves modified with Ni *in situ* were synthesized by the two-step crystallization method. In a typical example, first, 60.75 g of deionized water and 12.97 g of phosphoric acid were mixed to form a solution, and then, 11.0 g of pseudo-boehmite and a certain amount of Ni (NO_3_)_2_·6H_2_O were added to the solution and stirred for 2 h. Second, 3.873 g of di-n-propylamine (DPA) and 3.873 g of diisopropylamine (DIPA) were added and continuously stirred for 2 h. Third, 6.76 g of acid silica sol was added dropwise to the system and stirred vigorously for 2 h. Finally, 1.17 g of dodecyltrimethylammonium bromide (DTAB) was added and stirred for 1 h to form an initial gel with a molar composition of 1.0 Al_2_O_3_: 0.75 P_2_O_5_: 0.45 SiO_2_: *x* Ni(NO_3_)_2_ (*x*/Al_2_O_3_ = 1, 2, 3, and 4%): 0.5 DPA: 0.5 DIPA: 0.05 DTAB: 45 H_2_O. The initial gel was precrystallized at 90°C for 12 h and then crystallized at 190°C for 24 h. The solid products collected by filtration were washed to neutrality with deionized water, dried at 110°C overnight, and calcined at 600°C for 6 h to obtain the Ni-modified SAPO-11 molecular sieves. The Ni-modified SAPO-11 samples were denoted as *x*Ni-SAPO-11 (*x* = 1, 2, 3, and 4).

For comparison, a conventional SAPO-11 molecular sieve was obtained through a similar procedure used for synthesizing *x*Ni-SAPO-11 above, but without adding Ni(NO_3_)_2_·6H_2_O.

### Preparation of NiW-Supported Catalysts

All the SAPO-11 samples were pressed, crushed, and sieved to obtain particles of a 20–40 mesh in size. The NiW-supported catalysts were prepared by the incipient wetness impregnation method with an aqueous solution of Ni(NO_3_)_2_·6H_2_O and (NH_4_)_6_H_2_W_12_O_40_·xH_2_O and then dried at 110°C for 6 h and calcined at 500°C for 4 h after evaporation at room temperature overnight. For each catalyst, the loading concentration of NiO was 5%, and the loading concentration of WO_3_ was 15%. The obtained catalysts were denoted as NiW/*x*Ni-SAPO-11 (*x* = 1, 2, 3, and 4) and NiW/SAPO-11.

### Characterization

X-ray diffraction (XRD) characterization of the SAPO-11 samples was performed on a Bruker AXS D8 Advance X-ray diffractometer with Cu Kα radiation working under 40 kV and 40 mA and 2θ ranging from 5 to 90° at a scanning speed of 5°/min. Scanning electron microscopy (SEM) images of the SAPO-11 samples were obtained on an FEI Quanta 200 F field-emission environmental scanning electron microscope. N_2_ adsorption–desorption characterization was carried out on a Micromeritics ASAP 2020 analyzer. The specific surface area, micropore volume, and mesopore volume were calculated by the Brunauer-Emmett-Teller (BET) method, the de Boer t-plot method, and the Barrett-Joyner-Halenda (BJH) method, respectively. Temperature-programmed desorption of ammonia (NH_3_-TPD) characterization was carried out on a Micromeritics auto-chem 2920 instrument. The samples were heated at 600°C for 30 min in an Ar flow, and then, the Ar flow was switched to an ammonia flow and kept for 30 min when the samples were cooled to 70°C. The samples were purged with Ar for 2 h to remove the physically adsorbed ammonia, and the TPD signal was recorded using a thermal conductivity detector (TCD) from 70°C to 600°C with a heating rate of 10°C/min. Pyridine adsorbed infrared (Py-IR) spectra were recorded on a Nicolet 5700 spectrometer, and the samples with pyridine were evacuated at 200°C and 350°C, respectively. X-ray photoelectron spectroscopy (XPS) characterization of the sulfided catalysts was performed on a Thermo spectrometer using Al Kα radiation as the excitation light source. All spectra used the Al 2p peak at a binding energy of 74.6 eV to calibrate the binding energy scale. The XPS spectra of the sulfided catalysts were decomposed by fitting with XPS PEAK, and the deconvolution was realized by Gaussian-Lorentzian band shapes. High-resolution transmission electron microscopy (HRTEM) images of the sulfided catalysts were obtained on a JEM 2100 LaB_6_ transmission electron microscope. The average length and average stacking number of WS_2_ slabs were calculated by the following equation (Yu et al., 2012):

$$\mathrm{Average}\;\mathrm{slab}\;\mathrm{length}\;\bar{L}=\frac{\sum_{i=1}^{n}n_{i}l_{i}}{\sum_{i=1}^{n}n_{i}},$$ (1)

$$\mathrm{Average}\;\mathrm{Stacking}\;\mathrm{Number}\;\bar{N}=\frac{\sum_{i=1}^{n}n_{i}N_{i}}{\sum_{i=1}^{n}n_{i}},$$ (2)

where *l*_*i*_ is the length of the WS_2_ slabs, *n*_*i*_ is the number of the slabs with length *l*_*i*_, and *N*_*i*_ is the number of layers of the WS_2_ slabs. Based on the assumption that WS_2_ slabs are standard hexagons, the dispersion degree of the WS_2_ active phase was calculated by the following equation (Tao et al., 2014):

$$f_{W}=\frac{W_{edge}}{W_{total}}=\frac{\sum_{i=1}^{t}6\left(n_{i}-1\right)}{\sum_{i=1}^{t}\left(3n_{i}^{2}-3n_{i}+1\right)},$$ (3)

where *W*_*edge*_ denotes the W atoms located on the edge of the WS_2_ slabs, *W*_*total*_ denotes the total W atoms, *n*_*i*_ is the number of W atoms along one side of a WS_2_ slab determined from its length [*L* = 3.2 (2*n*_*i*_−1) Å], and *t* is the total number of slabs determined by no less than 500 WS_2_ slabs obtained from the HRTEM images of different parts of the different catalysts.

### Catalytic Performance Assessment

The hydroisomerization of n-hexadecane (n-C_16_) was carried out in a fixed-bed hydrogenation microreactor. In a typical example, 3 g of the catalyst was packed in the middle of the reaction tube, and both ends were filled with silica sand. Before the reaction, the catalyst was presulfurized with cyclohexane solution containing 2 vol% CS_2_ for 5 h at 320°C, a liquid hourly space velocity (LHSV) of 7 h^−1^, and H_2_/oil of 100 (v/v). After the presulfurization treatment was completed, the catalytic performance of the catalyst was investigated under the conditions of a reaction pressure of 2 MPa, an LHSV of 1.5 h^−1^, H_2_/oil of 600 (v/v), and a reaction temperature of 320–400°C. The reactant n-hexadecane was fed into the reactor using a syringe pump when the temperature was decreased to the reaction temperature. The products collected were analyzed on a Shimadzu GC-2014 gas chromatograph equipped with a capillary HP-PONA column, and the qualitative analysis of the products was realized by GC-MS. The conversion of n-C_16_ of each catalyst at 340°C was converted into the TOF (turnover frequency, which is used to evaluate the catalytic activity of the catalyst) of each active site, which was calculated by the following equation based on the number of all available active sites (Wen et al., 2019):

$$TOF=\frac{V_{feed}\cdot x}{n_{W}\cdot f_{W}},$$ (4)

where *V*_*feed*_ is the feed flow rate of the reactant n-C_16_ in mol/h, *x* is the conversion of n-C_16_ at 340°C, *n*_*w*_ is the amount of W atoms of the catalyst in mol, and *f*_*W*_ is the dispersion degree of W species.

## Results and Discussion

### Crystalline Phase

**FIGURE 1:**
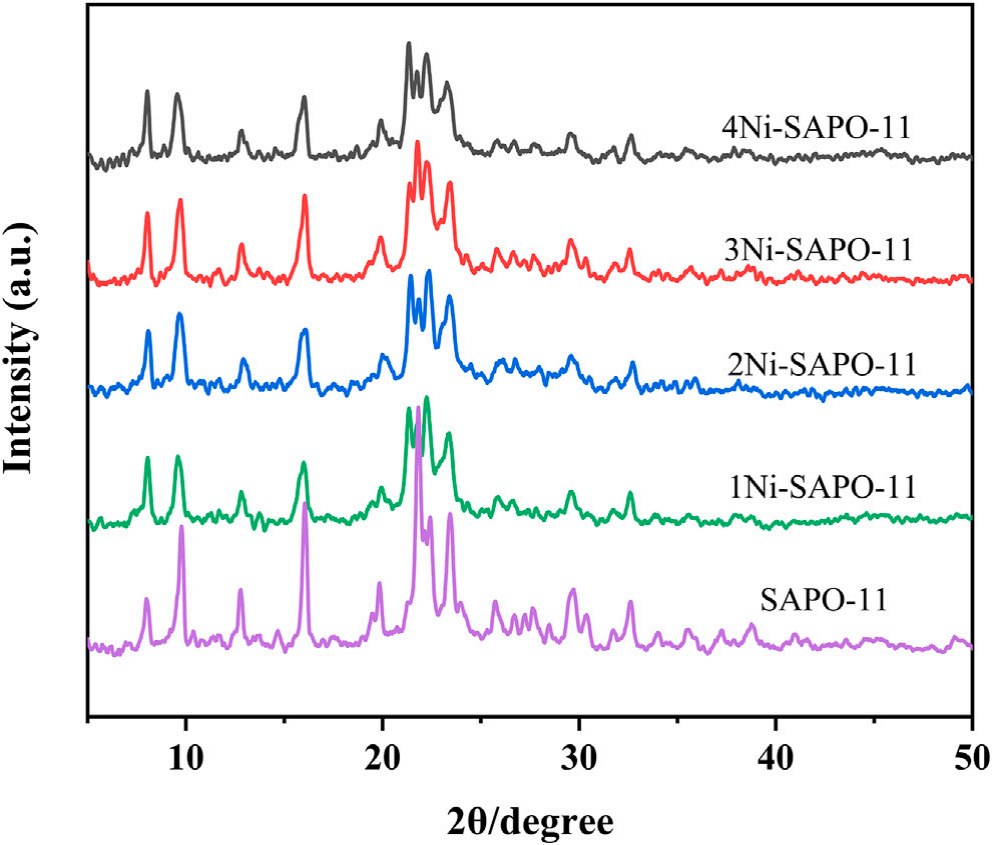
XRD patterns of Ni-modified SAPO-11 molecular sieves.

Figure 1 presents the X-ray diffraction (XRD) patterns of the Ni-modified SAPO-11 molecular sieves. It can be seen that all the Ni-modified SAPO-11 samples presented characteristic diffraction peaks at 2θ = 8.2°, 9.6°, 13.0°, 15.8°, 20.2°, 21.1°, and 22.0–23.3°, which are attributed to the typical AEL structure, and no characteristic diffraction peaks of other crystalline phases were observed (Jin et al., 2016). The results indicate that the crystal structural units of the Ni-modified SAPO-11 molecular sieves were completely preserved. In addition, no characteristic diffraction peaks of Ni species were observed in the XRD patterns of all the Ni-modified SAPO-11 samples, indicating that Ni species were highly dispersed in SAPO-11. This result can be explained by the fact that Ni species substituted some of the Al species and entered the framework structure of SAPO-11 in the process of *in situ* modification.

### Morphology

**FIGURE 2:**
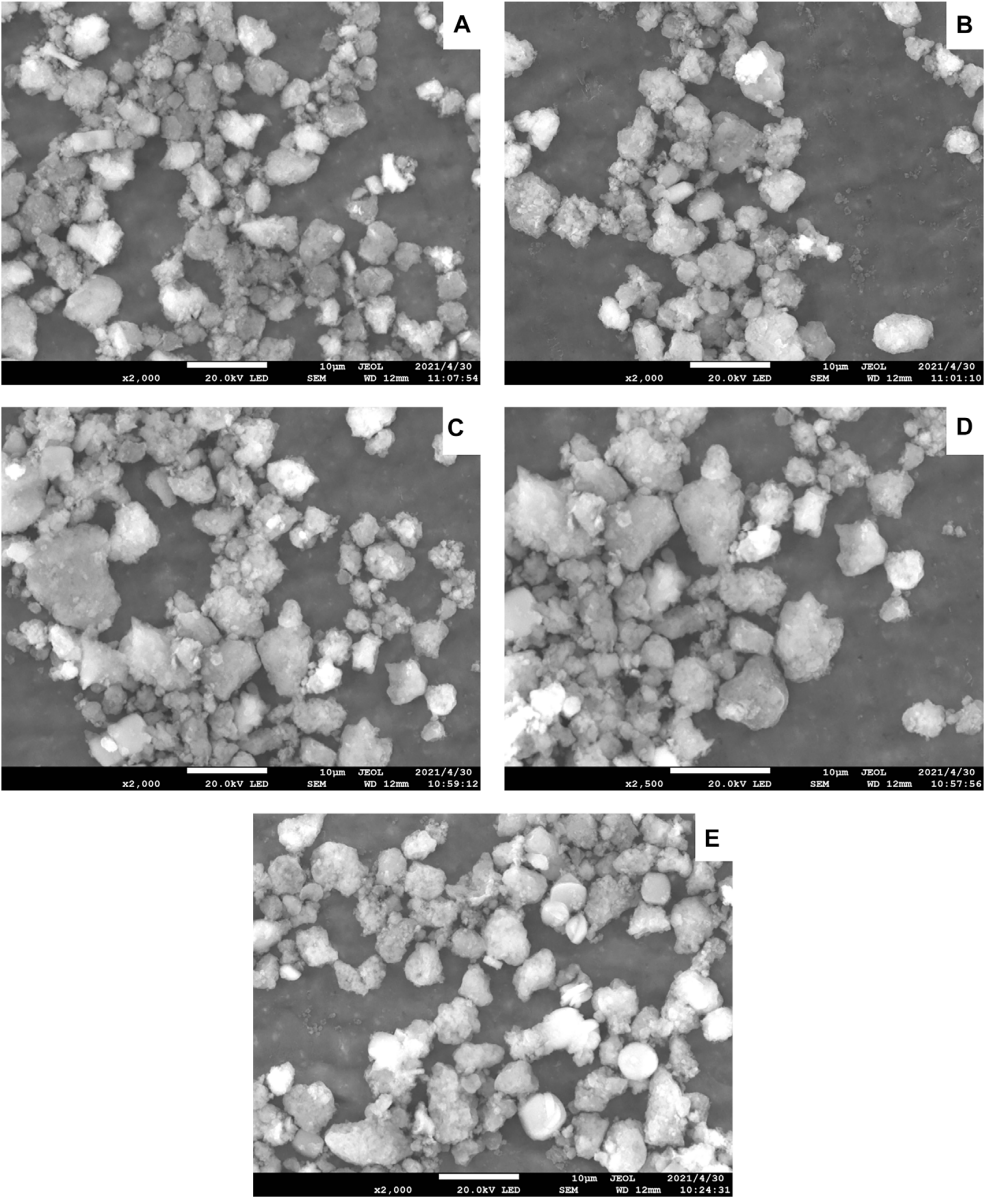
SEM images of Ni-modified SAPO-11 molecular sieves: **(A)** 4Ni-SAPO-11, **(B)** 3Ni-SAPO-11, **(C)** 2Ni-SAPO-11, **(D)** 1Ni-SAPO-11, and **(E)** SAPO-11.

The SEM images of the Ni-modified SAPO-11 samples are presented in Figure 2. All the samples showed pseudo-spherical particles with a particle size of about 3–7 μm, indicating that the introduction of Ni did not change the particle size distribution of SAPO-11 in the synthesis process. Moreover, no amorphous phase was observed in SEM images of all samples, indicating that the introduction of Ni into SAPO-11 by *in situ* modification did not change the morphology of SAPO-11. Through further careful observation, it can be found that the particles of all samples were assembled by many crystallites, which provides conditions for the formation of more intercrystallite mesoporous structures.

### Textural Properties

**FIGURE 3:**
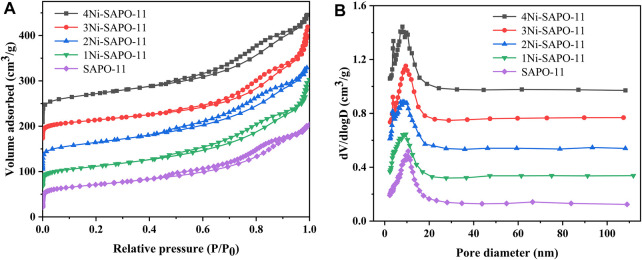
N_2_ adsorption–desorption isotherms **(A)** and pore size distribution **(B)** of Ni-modified SAPO-11 molecular sieves.

**FIGURE 4:**
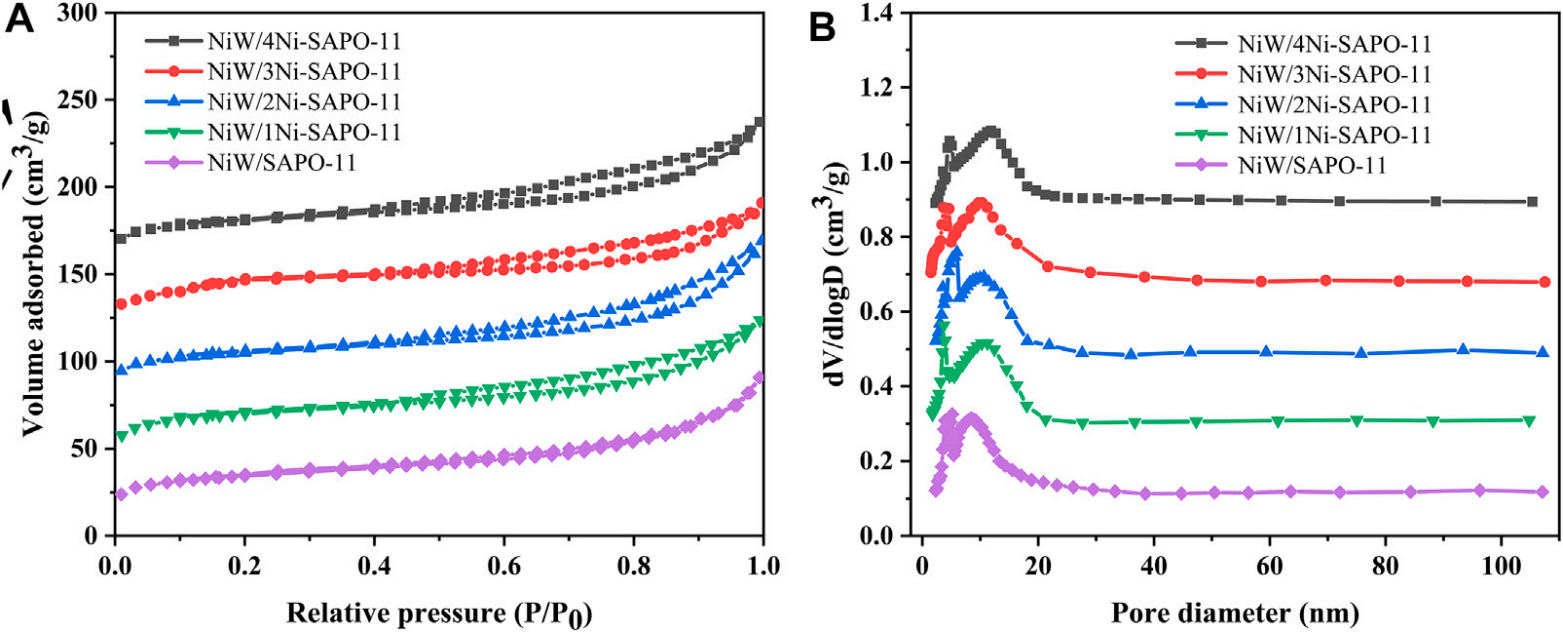
N_2_ adsorption–desorption isotherms **(A)** and pore size distribution **(B)** of different catalysts.

In order to obtain the textural properties such as specific surface area, pore size, and pore volume of the Ni-modified SAPO-11 molecular sieves, N_2_ adsorption–desorption tests of all the samples were carried out. The N_2_ adsorption–desorption isotherms and pore size distribution curves of all the samples are presented in Figure 3, while the data of texture properties are listed in Table 1. As shown in Figure 3A, all the samples showed type IV isotherms and typical H4-type hysteresis loops (Verma et al., 2015; Tao et al., 2017). The N_2_ adsorption amount of all the samples increased sharply at a lower relative pressure (10^–5^ < P/P_0_ < 10^–2^), which was attributed to the adsorption of N_2_ in micropores (Liu et al., 2015). All the samples showed obvious hysteresis loops in the range of relative pressures of 0.4–0.9, indicating that there were a large number of mesoporous structures in all the samples. In addition, the hysteresis loops of Ni-modified SAPO-11 samples were larger than those of the unmodified SAPO-11, indicating that Ni-modified SAPO-11 samples possessed more mesoporous structures. Figure 3B presented the pore size distribution curves of all the samples, and it can be seen that the pore size of all samples was mainly distributed between 6 and 10 nm. Table 1 lists the textural property data of the Ni-modified SAPO-11 samples. The BET specific surface area (S_BET_) of Ni-modified SAPO-11 samples increased in the order of SAPO-11 (166 m^2^/g) < 1Ni-SAPO-11 (169 m^2^/g) < 2Ni-SAPO-11 (171 m^2^/g) < 3Ni-SAPO-11 (174 m^2^/g) < 4Ni-SAPO-11 (176 m^2^/g) and the mesopore volume (V_meso_) of Ni-modified SAPO-11 samples also increased in the order of SAPO-11 (0.35 m^3^/g) < 1Ni-SAPO-11 (0.36 m^3^/g) < 2Ni-SAPO-11 (0.37 m^3^/g) < 3Ni-SAPO-11 (0.39 m^3^/g) < 4Ni-SAPO-11 (0.41 m^3^/g), which indicate that Ni *in situ* modification is beneficial to increase the S_BET_ and V_meso_ of SAPO-11. Figure 4 and Table 2 present the N_2_ adsorption–desorption isotherms, pore size distribution, and textural data of the different catalysts, respectively. It is not difficult to see that the pore size distribution of the catalysts supported with NiW is basically unchanged. However, the BET specific surface area, external specific surface area, micropore volume, and mesopore volume of the catalysts all decreased, and the change order of these data of the different catalysts still followed the change order of these data of the Ni-modified SAPO-11 molecular sieves.

**TABLE 1:** Textural properties of Ni-modified SAPO-11.

Sample	S_BET_, m^2^/g	S_ext_, m^2^/g	V_micro_, cm^3^/g	V_meso_, cm^3^/g	V_total_, cm^3^/g
4Ni-SAPO-11	176	98	0.08	0.33	0.41
3Ni-SAPO-11	174	94	0.07	0.32	0.39
2Ni-SAPO-11	171	95	0.08	0.29	0.37
1Ni-SAPO-11	169	89	0.06	0.30	0.36
SAPO-11	166	91	0.09	0.26	0.35

**TABLE 2:** Textural properties of Ni-modified SAPO-11.

Sample	S_BET_, m^2^/g	S_ext_, m^2^/g	V_micro_, cm^3^/g	V_meso_, cm^3^/g	V_total_, cm^3^/g
NiW/4Ni-SAPO-11	160	91	0.07	0.30	0.37
NiW/3Ni-SAPO-11	158	87	0.05	0.28	0.33
NiW/2Ni-SAPO-11	153	84	0.06	0.26	0.32
NiW/1Ni-SAPO-11	149	79	0.05	0.25	0.30
NiW/SAPO-11	145	82	0.07	0.23	0.30

### Acidity Properties

**FIGURE 5:**
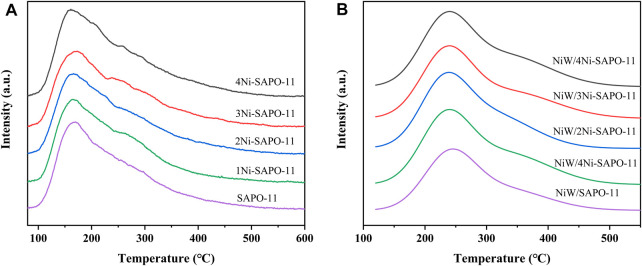
NH_3_-TPD profiles of Ni-modified SAPO-11 molecular sieves **(A)** and different catalysts **(B)**.

Figure 5 presents the temperature-programmed desorption of ammonia (NH_3_-TPD) profiles of Ni-modified SAPO-11 samples. Each curve can be fitted and deconvolved into three peaks with a Gaussian function. The three decomposition peaks near 180°C, 250°C, and 330°C correspond to the desorption of NH_3_ adsorbed on weak acid sites, medium acid sites, and strong acid sites, respectively (Liu et al., 2009; Liu et al., 2020). It can be seen that with the increase of Ni content in the process of Ni modification, the intensity of low-temperature desorption peaks of the Ni modified SAPO-11 samples gradually decreased, indicating that the weak acid amount of Ni-modified SAPO-11 samples decreased with the increase of Ni content. The intensity of medium-temperature desorption peaks and high-temperature desorption peaks of Ni-modified SAPO-11 samples increased in the order of SAPO-11 < 1Ni-SAPO-11 < 2Ni-SAPO-11 < 4Ni-SAPO-11 < 3Ni-SAPO-11, indicating that the medium and strong acid amounts also increased in the same order. This result can be explained by the fact that the average electronegativity of Ni (1.91) is higher than that of Al (1.71), and the covalency of Ni is higher than that of Al (Zhou et al., 2017). Therefore, the Brønsted protons and Lewis caves of SAPO-11 formed by substituting part of Al species with Ni species have stronger acid density. However, when the Ni content reached 4%, not only Ni species entered the framework of SAPO-11 but also excessive nickel species located on the surface of SAPO-11 and covered a part of acid sites. Therefore, the medium and strong acid amounts of 4Ni-SAPO-11 are less than that of 3Ni-SAPO-11. In addition, the different catalysts were also characterized by NH_3_-TPD. Compared with Ni-modified SAPO-11 molecular sieves, the peak areas of NH_3_-TPD profiles of the catalysts all decreased, indicating that the acid amount of the catalysts decreased after being supported with NiW. However, the variation in peak area of the catalysts was still consistent with that of the Ni-modified SAPO-11 samples.

**FIGURE 6:**
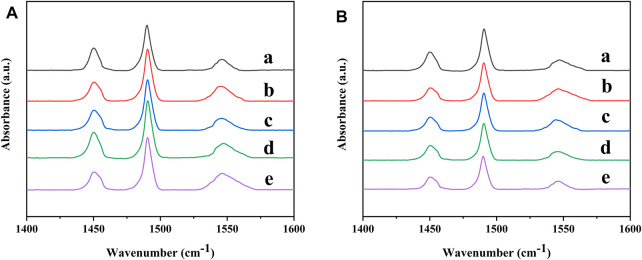
Py-IR spectra of Ni-modified SAPO-11 molecular sieves: (a) 4Ni-SAPO-11, (b) 3Ni-SAPO-11, (c) 2Ni-SAPO-11, (d) 1Ni-SAPO-11, and (e) SAPO-11 at 200°C **(A)** and 350°C **(B)**.

To qualitatively analyze the acidity of the Ni-modified SAPO-11 samples, the samples were characterized by Py-IR. The Py-IR spectra of the Ni-modified SAPO-11 samples are presented in Figure 6. The weak acid amount was calculated from the Py-IR spectra formed by pyridine molecules desorbed at 200°C (Figure 6A), while the medium and strong acid amounts were calculated from the Py-IR spectra formed by pyridine molecules desorbed at 350°C (Figure 6B). The corresponding acid amount data are listed in Table 3. As shown in Figure 6, all the samples showed three peaks in the range of 1,400–1,600 cm^−1^. The two peaks at 1,455 cm^−1^ and 1,545 cm^−1^ correspond to the desorption of pyridine molecules on Brønsted (B) acid sites and Lewis (L) acid sites, respectively, while the peak at 1,490 cm^−1^ is attributed to the synergistic effect of B acid sites and L acid sites (Guo et al., 2013; Du et al., 2019). Compared with SAPO-11, the weak L acid amount of Ni-modified SAPO-11 samples increased and the weak B acid amount decreased. This result can be explained by the fact that NiO entering the framework of SAPO-11 can form some new weak L acid sites in the process of Ni *in situ* modification. The weak B acid sites of SAPO-11 are generated by Si-OH, Al-OH, and P-OH (Yang et al., 2017). Ni species substitute some Al species to form Ni-OH species, which cannot form weak B acid sites like Al-OH species. The amount of medium and strong B acid sites of the Ni-modified SAPO-11 samples increased in the order of SAPO-11 (30.73 μmol/g) < 1Ni-SAPO-11 (32.42 μmol/g) < 4Ni-SAPO-11 (32.62 μmol/g) < 2Ni-SAPO-11 (35.25 μmol/g) < 3Ni-SAPO-11 (37.21 μmol/g). The result can be explained by the fact that the medium and strong B acid sites of SAPO-11 are generated by the Si-OH-Al species (Blasco et al., 2006). In the process of Ni *in situ* modification, Ni species substitute some Al species to form Si-OH-Ni species, and their acid density is greater than that of Si-OH-Al species. The reason for the medium and strong B acid amounts of 4Ni-SAPO-11 being lower than that of 3Ni-SAPO-11 is that excessive nickel species could not completely enter the framework of 4Ni-SAPO-11 and were distributed on the surface and pores of SAPO-11 and covered some acid sites. The results of the acid amount reflected by Py-IR tests well verified the results of the acid amount reflected by NH_3_-TPD.

**TABLE 3:** Acidity properties of Ni-modified SAPO-11 determined by Py-IR.

Sample	Acidity (μmol/g)					
	Weak acid sites (200°C)			Medium and strong acid sites (350°C)		
	B	L	B+ L	B	L	B+ L
4Ni-SAPO-11	59.87	37.32	97.19	32.62	22.97	55.59
3Ni-SAPO-11	61.91	37.84	99.75	37.21	21.16	58.37
2Ni-SAPO-11	69.26	38.45	107.71	35.25	21.61	56.86
1Ni-SAPO-11	82.04	39.5	121.54	32.42	21.89	54.31
SAPO-11	91.18	37.04	128.22	30.73	19.46	50.19

### Active Phase Characterization

**FIGURE 7:**
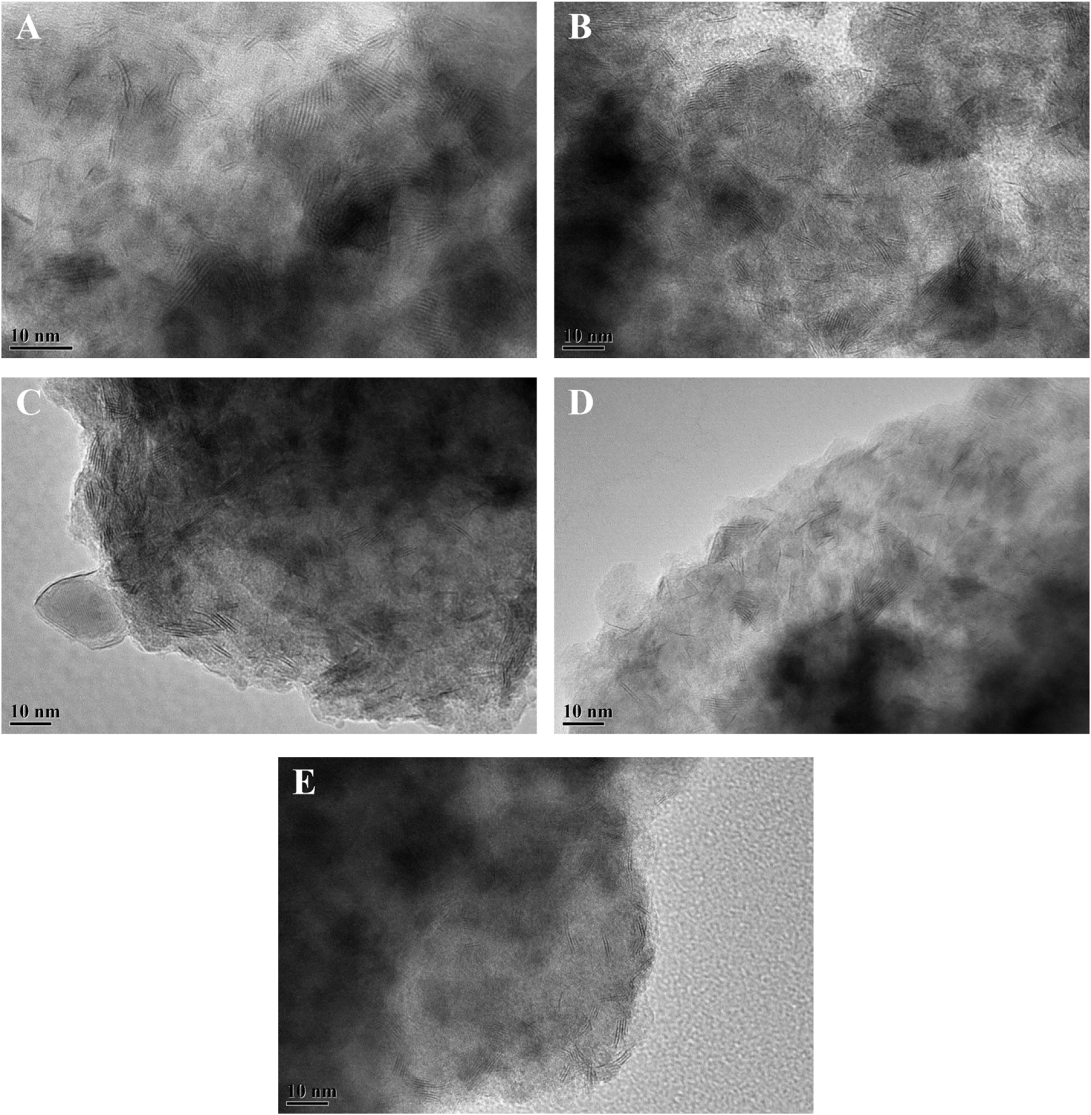
Representative HRTEM images of sulfided catalysts: **(A)** NiW/4Ni-SAPO-11, **(B)** NiW/3Ni-SAPO-11, **(C)** NiW/2Ni-SAPO-11, **(D)** NiW/1Ni-SAPO-11, and **(E)** NiW/SAPO-11.

To visualize the morphology and stacking of the active phase on the catalysts, HRTEM characterization of the sulfided catalysts was carried out. The representative HRTEM images of the different sulfided catalysts are presented in Figure 7. The black line-like layers in the images are the typical WS_2_ slabs, and the NiS_x_ phase is too small to be observed in the images. The statistical analysis was carried out on no less than 500 slabs from different parts of different catalysts. The average length and average stacking number of WS_2_ slabs on each catalyst were calculated from Eqs 1, 2, respectively, and the results are listed in Table 4. The statistical results showed that the average length of WS_2_ slabs on different catalysts increased in the order of NiW/3Ni-SAPO-11 (2.98 nm) < NiW/4Ni-SAPO-11 (3.23 nm) < NiW/2Ni-SAPO-11 (3.46 nm) < NiW/1Ni-SAPO-11 (4.03 nm) < NiW/SAPO-11 (4.72 nm). The length of WS_2_ slabs on NiW/SAPO-11 was in the range of 2–8 nm and mainly concentrated in 3–7 nm. The lengths of WS_2_ slabs on NiW/1Ni-SAPO-11, NiW/2Ni-SAPO-11, NiW/3Ni-SAPO-11, and NiW/4Ni-SAPO-11 were mainly concentrated in 2–7 nm, 2–6 nm, 1–4 nm, and 2–5 nm, respectively. The results showed that Ni *in situ* modification decreased the slab length of the active phase. It is believed that the length of active phase slabs is related to the dispersion of the active phase. The longer the length of active phase slabs, the lower the dispersion degree of the active phase (Zhou et al., 2018). The result indicates that the dispersion degree of the so-called NiWS active phase decreased in the order of NiW/3Ni-SAPO-11 > NiW/4Ni-SAPO-11 > NiW/2Ni-SAPO-11 > NiW/1Ni-SAPO-11 > NiW/SAPO-11. The stacking number of the active phase can reflect the interaction between the active phase and support to a certain extent. Generally speaking, the higher the stacking number of the active phase, the weaker the interaction between the active phase and support (Cui et al., 2012). The average stacking number of WS_2_ slabs on the different catalysts increased in the order of NiW/SAPO-11 (1.33) < NiW/1Ni-SAPO-11 (1.85) < NiW/2Ni-SAPO-11 (2.68) < NiW/4Ni-SAPO-11 (3.06) < NiW/3Ni-SAPO-11 (3.23), indicating that the interaction between the active phase and support decreased in the order of NiW/SAPO-11 > NiW/1Ni-SAPO-11 > NiW/2Ni-SAPO-11 > NiW/4Ni-SAPO-11 > NiW/3Ni-SAPO-11. In order to further verify the change of dispersion degree of the active phase on the different catalysts, the dispersion degree of tungsten species (*f*_*W*_) on the different catalysts was calculated by Equation 3. The results showed that the dispersion degree of tungsten species on different catalysts decreased in the order of NiW/3Ni-SAPO-11 > NiW/2Ni-SAPO-11 > NiW/4Ni-SAPO-11 ≈ NiW/1Ni-SAPO-11 > NiW/SAPO-11, which was completely consistent with the results reflected by the length and stacking number of WS_2_ slabs. The result can be explained by the fact that Al in W-O-Al species was substituted by Ni to form W-O-Ni species on the Ni-modified SAPO-11-supported NiW catalysts. However, the W-O-Ni bond is weaker than the W-O-Al bond, which weakened the interaction between the active phase and support and made the dispersion of the active phase poor. However, the average slab length of the active phase of NiW/4Ni-SAPO-11 was longer than that of NiW/3Ni-SAPO-11, and the stacking number of the active phase of NiW/4Ni-SAPO-11 was lower than that of NiW/3Ni-SAPO-11. This may be due to the fact that excessive nickel species did not completely enter the framework of the SAPO-11 molecular sieve and distributed on the surface and pores of SAPO-11, which hindered the dispersion of the so-called NiWS active phase.

**TABLE 4:** Average lengths, layer numbers, and *f*_*W*_ values of WS_2_ of all catalysts.

Sample	$\bar{L}$ (nm)	$\bar{N}$	*f*_*W*_
NiW/4Ni-SAPO-11	3.23	3.09	0.29
NiW/3Ni-SAPO-11	2.98	3.23	0.31
NiW/2Ni-SAPO-11	3.46	2.68	0.30
NiW/1Ni-SAPO-11	4.03	1.85	0.29
NiW/SAPO-11	4.72	1.33	0.28

**FIGURE 8:**
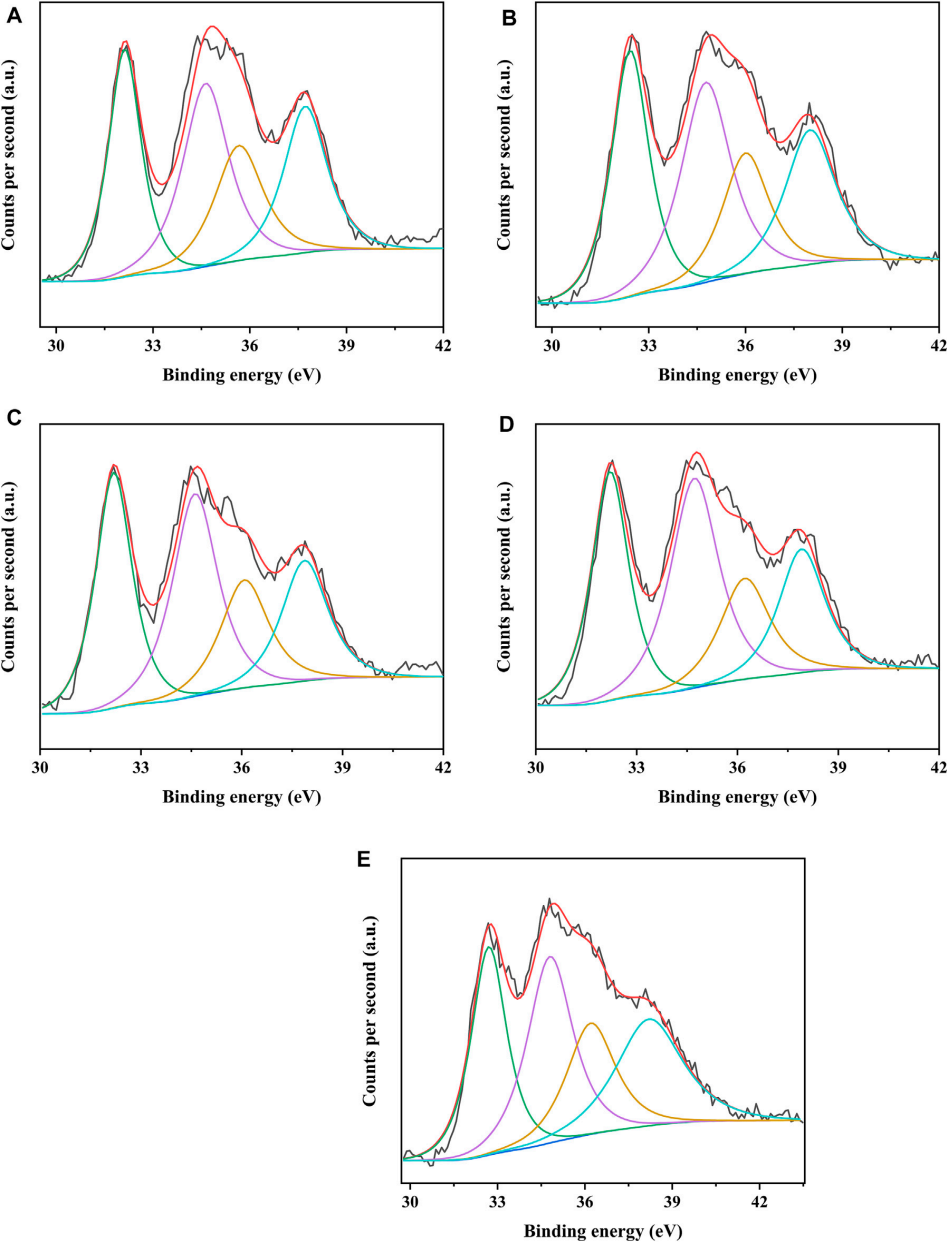
XPS W 4f spectra of sulfided catalysts: **(A)** NiW/4Ni-SAPO-11, **(B)** NiW/3Ni-SAPO-11, **(C)** NiW/2Ni-SAPO-11, **(D)** NiW/1Ni-SAPO-11, and **(E)** NiW/SAPO-11.

**FIGURE 9:**
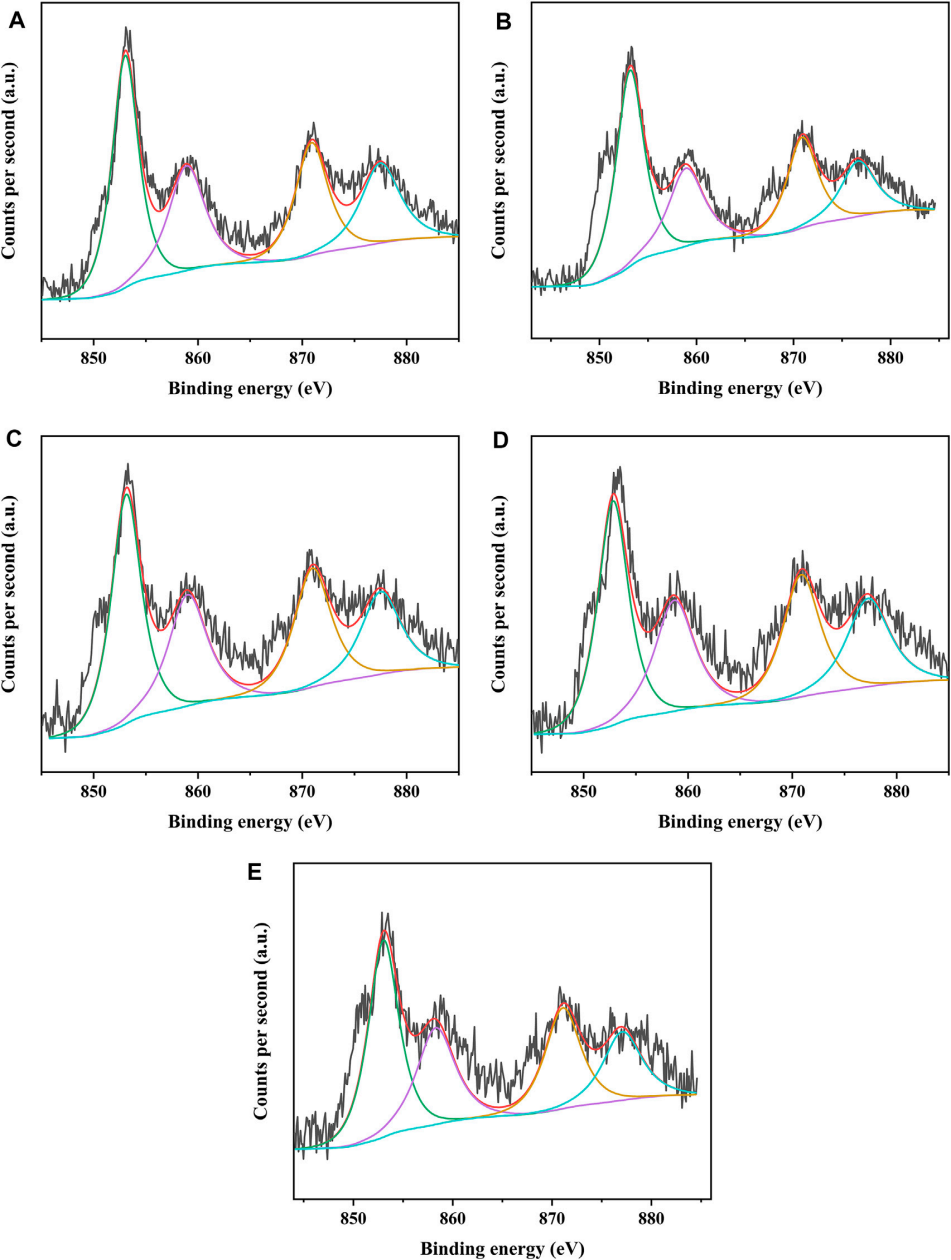
XPS Ni 2p spectra of sulfided catalysts: **(A)** NiW/4Ni-SAPO-11, **(B)** NiW/3Ni-SAPO-11, **(C)** NiW/2Ni-SAPO-11, **(D)** NiW/1Ni-SAPO-11, and **(E)** NiW/SAPO-11.

The different catalysts were characterized by XPS to analyze the covalent state of nickel species and tungsten species on the catalysts. Figures 8, 9 present the W4f XPS spectra and Ni2p XPS spectra of the sulfided catalysts, respectively. The sulfidation degree of tungsten species and nickel species was calculated by the deconvolution peak area. The results of the binding energy (BE) and sulfidation degree of tungsten species and nickel species of the sulfided catalysts are listed in Tables 5, 6, respectively. The W 4f XPS spectra of different catalysts were decomposed into four peaks, which were attributed to the two overlapping peaks of W^4+^ and W^6+^. The decomposition peaks in Figure 8 with binding energies of 36.30 ± 0.40 eV and 38.10 ± 0.40 eV were attributed to the W 4f_7/2_ levels and W 4f_5/2_ levels of W^6+^ (WO_3_), respectively, while the decomposition peaks with binding energies of 32.40 ± 0.40 eV and 34.80 ± 0.40 eV were attributed to the W 4f_7/2_ levels and W 4f_5/2_ levels of W^4+^ (WS_2_), respectively. The binding energies of W 4f_7/2_ levels and W 4f_5/2_ levels of the investigated catalysts decreased in the order of NiW/SAPO-11 > NiW/1Ni-SAPO-11 > NiW/2Ni-SAPO-11 > NiW/3Ni-SAPO-11 > NiW/4Ni-SAPO-11, which indicates that the interaction between the active phase and support of the investigated catalysts also decreased in the same order, which was consistent with the results reflected by HRTEM images. The sulfidation degree of the tungsten species of different catalysts was defined as W^4+^/(W^4+^ + W^6+^) (Cui et al., 2013), which increased in the order of NiW/SAPO-11 (56.29%) < NiW/4Ni-SAPO-11 (57.23%) < NiW/1Ni-SAPO-11 (57.69%) < NiW/2Ni-SAPO-11 (58.48%) < NiW/3Ni-SAPO-11 (60.93). This result can be explained by the following facts: Ni species substituted some Al species, resulting in some of the W-O-Al bonds being substituted with W-O-Ni bonds in the process of Ni *in situ* modification, and the strength of W-O-Ni bonds was lower than that of W-O-Al bonds, thus reducing the interaction between the active phase and support and improving the sulfidation degree of tungsten species. However, the excessive addition of Ni in the process of *in situ* modification of SAPO-11 led to poor dispersion of active metals on the catalyst, which led to the lower sulfidation degree of tungsten species of NiW/4Ni-SAPO-11 than NiW/3Ni-SAPO-11. The Ni 2p XPS spectra of different catalysts could also be decomposed into four peaks, which were attributed to the two overlapping peaks of nickel oxide and nickel sulfide. The decomposition peaks in Figure 9 with binding energies of 858.80 ± 0.40 eV and 877.40 ± 0.40 eV were attributed to the Ni 2p_3/2_ levels and Ni 2p_1/2_ levels of nickel oxide (NiO), respectively, while the decomposition peaks with binding energies of 853.20 ± 0.40 eV and 870.70 ± 0.40 eV were attributed to the Ni 2p_3/2_ levels and Ni 2p_1/2_ levels of nickel sulfide (NiS_x_), respectively. The decreasing order of the binding energy of the levels of Ni is the same as that of the levels of W described above. This result further confirms that Ni *in situ* modification weakened the interaction between the active phase and support. The sulfidation degree of the nickel species was defined as NiS_x_/(NiO + NiS_x_) (Cui et al., 2013), which increased in the order of NiW/SAPO-11 (60.17%) < NiW/1Ni-SAPO-11 (60.69%) < NiW/2Ni-SAPO-11 (61.58%) < NiW/4Ni-SAPO-11 (62.53%) < NiW/3Ni-SAPO-11 (64.93). The reason for this result is the same as the above explanation about tungsten species.

**TABLE 5:** Binding energy and sulfidation degree of W on different catalysts.

Sample	NiW/4Ni-SAPO-11	NiW/3Ni-SAPO-11	NiW/2Ni-SAPO-11	NiW/1Ni-SAPO-11	NiW/SAPO-11
Oxidic	Binding energy (eV)				
W 4f_7_	35.66	36.00	36.08	36.22	36.50
W4f_5_	37.72	37.80	37.87	37.91	38.43
Sulfided	Binding energy (eV)				
W 4f_7_	32.12	32.19	32.21	32.24	32.80
W4f_5_	34.63	34.67	34.71	34.72	35.10
Sulfidation degree of W (%)	57.23	60.93	58.48	57.69	56.29

**TABLE 6:** Binding energy and sulfidation degree of Ni on different catalysts.

Sample	NiW/4Ni-SAPO-11	NiW/3Ni-SAPO-11	NiW/2Ni-SAPO-11	NiW/1Ni-SAPO-11	NiW/SAPO-11
Oxidic	Binding energy (eV)				
Ni 2p_3_	858.60	858.75	858.80	858.89	858.99
Ni 2p_1_	877.47	876.66	877.11	877.16	877.27
Sulfided	Binding energy (eV)				
Ni 2p_3_	853.02	853.14	853.22	853.31	853.43
Ni 2p_1_	870.58	870.68	870.77	870.86	871.09
Sulfidation degree of Ni (%)	62.53	64.93	61.58	60.69	60.17

### Catalytic Performance

**FIGURE 10:**
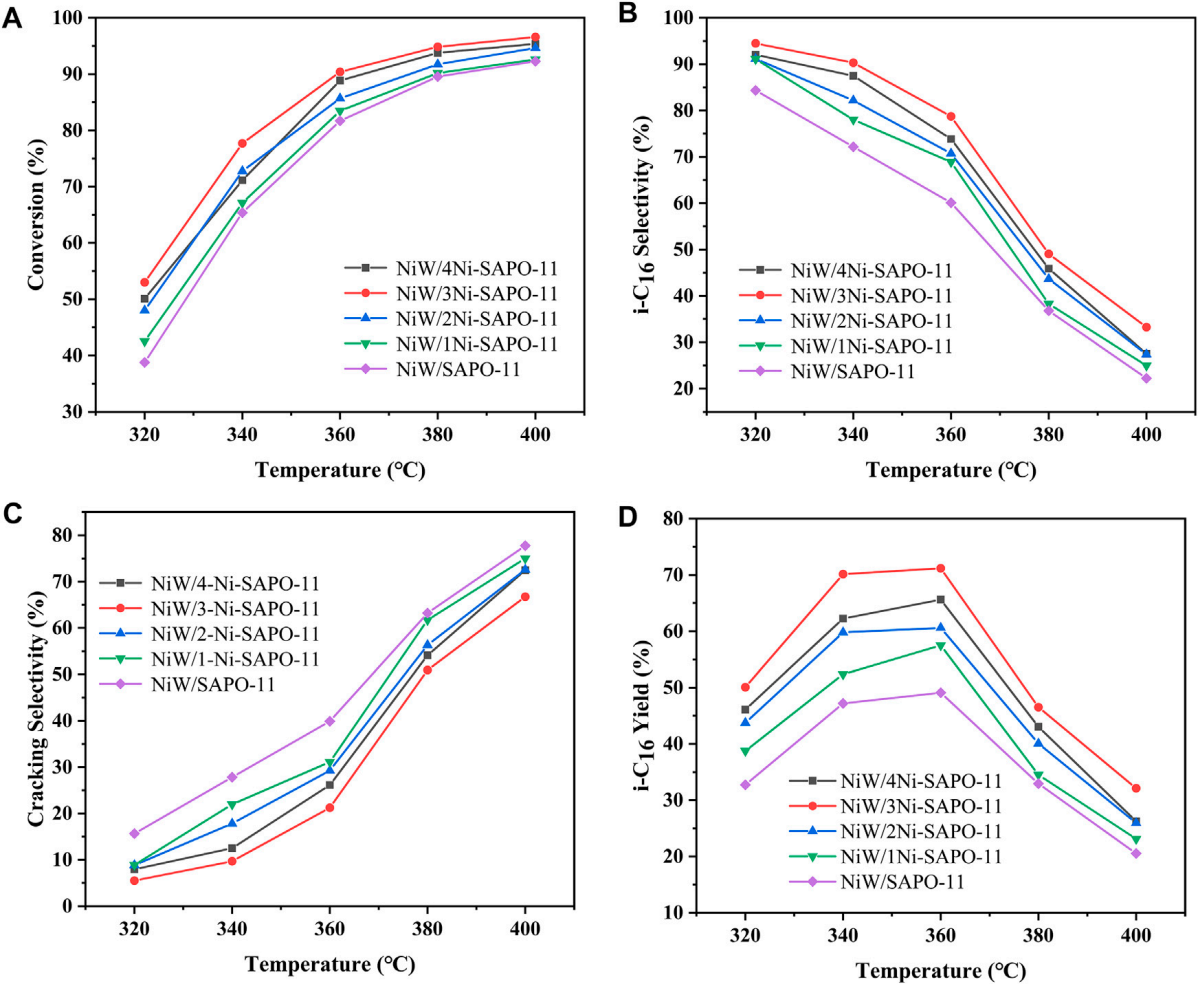
Catalytic performance of the different catalysts: **(A)** n-C_16_ conversion, **(B)** i-C_16_ selectivity, **(C)** cracking selectivity, and **(D)** i-C_16_ yield.

The evaluation of the hydroisomerization performance of all the catalysts was carried out in a fixed-bed hydrogenation microreactor. In each reaction, the catalyst was presulfurized in advance, the reaction pressure was fixed at 2.0 MPa, the liquid hourly space velocity (LHSV) was fixed at 1.5 h^−1^, and H_2_/oil was 600 (v/v). The catalytic performance of the catalysts was evaluated in the range of 320–400°C. Figure 10A presents the conversion of n-C_16_ on the different catalysts at different reaction temperatures. It can be seen that the n-C_16_ conversion of all the catalysts increased with the increase of temperature. Among all the investigated catalysts, NiW/3Ni-SAPO-11 showed the highest conversion of n-C_16_ in the range of investigated reaction temperatures, which indicates that NiW/3Ni-SAPO-11 had the highest catalytic activity among all the investigated catalysts, and the catalytic activities of the catalysts increased in the order of NiW/SAPO-11 < NiW/1Ni-SAPO-11 < NiW/2Ni-SAPO-11 < NiW/4Ni-SAPO-11 < NiW/3Ni-SAPO-11. The result can be explained by the fact that the amount of acid in the support and the properties of the active phase jointly determine the catalytic activity of the catalyst. It is generally believed that the acid amount of the catalyst, especially the medium and strong B acid amounts, determines the isomerization rate of olefin intermediates in the isomerization process. Moreover, the higher the dispersion degree of the active phase, the better the hydrogenation performance of the catalyst, and with the appropriate increase of the number of stacking layers of the active phase, the hydrogenation performance of the active phase also increases. The appropriate interaction between the active phase and support of NiW/3Ni-SAPO-11 and the highest dispersion of the active phase made NiW/3Ni-SAPO-11 show the highest catalytic activity. The selectivity of isomeric hexadecane (i-C_16_) decreased with the increase of reaction temperature, which indicates that the increase of reaction temperature intensified the cracking reaction. The selectivity of i-C_16_ decreased greatly when the reaction temperature reached 380°C. Among all the investigated catalysts, the i-C_16_ selectivity of NiW/3Ni-SAPO-11 was the highest in the range of investigated reaction temperatures, and the i-C_16_ selectivity of different catalysts at different reaction temperatures basically increased in the order of NiW/SAPO-11 < NiW/1Ni-SAPO-11 < NiW/2Ni-SAPO-11 < NiW/4Ni-SAPO-11 < NiW/3Ni-SAPO-11 (Figure 10B). This result can be explained by the fact that 3Ni-SAPO-11 possessed the most medium and strong B acid sites, which are considered as the active centers of the isomerization of the alkane skeleton (Lyu et al., 2017). Meanwhile, the increase of mesoporous structures alleviated the diffusion resistance of n-hexadecane and isomeric hexadecane in the channels of SAPO-11, thus reducing the occurrence of cracking reactions. 3Ni-SAPO-11 showed the highest selectivity of i-C_16_ due to its highest medium and strong B acid amount and mesopore volume. Among all the investigated catalysts, the i-C_16_ yield of NiW/3Ni-SAPO-11 was the highest in the range of investigated temperatures, and the highest yield was 71.18% (Figure 10D).

Table 7 lists the isomer distributions and TOF values of the different catalysts at 340°C. It is not difficult to find that the isomer products of all the catalysts are mainly monobranched isomers and double-branched isomers, and the selectivity of monobranched isomers is much higher than that of double-branched isomers, and the selectivity of monomethyl branched isomers is much higher than that of monoethyl branched isomers. Among the isomer products, monobranched isomers mainly include 2-methylpentadecane (2-MC_15_), 3-methylpentadecane (3-MC_15_), 4-methylpentadecane (4-MC_15_), 5-methylpentadecane (5-MC_15_), 6-methylpentadecane (6-MC_15_), 3-ethyltetradecane (3-EC_14_), 5-ethyltetradecane (5-EC_14_), and 6-ethyltetradecane (6-EC_14_), while the double-branched isomers mainly include 2,4-dimethyltetradecane (2,4-DMC_14_), 2,5-dimethyltetradecane (2,5-DMC_14_), 2,6-dimethyltetradecane (2,6-DMC_14_), 3,6-dimethyltetradecane (3,6-DMC_14_), 3,7-dimethyltetradecane (3,7-DMC_14_), and 5,8-diethyldodecane (5,8-DEC_12_). Among all the isomer products, the selectivity of 2-MC_15_ and 3-MC_15_ is obviously higher than that of other isomer products, which is the same as that reported by Zhang et al. (Zhang et al., 2018). TOF values of the different catalysts refer to the standardized turnover frequency at each available active phase center, which can effectively reflect the catalytic activity of the catalysts. TOF of the different catalysts increased in the order of NiW/SAPO-11 (27.70 h^−1^) > NiW/4Ni-SAPO-11 (27.71 h^−1^) > NiW/1Ni-SAPO-11 (28.44 h^−1^) > NiW/2Ni-SAPO-11 (29.80 h^−1^) > NiW/3Ni-SAPO-11 (30.81 h^−1^), which well verified the results reflected by the above catalytic activity.

**TABLE 7:** Isomer distributions and TOF values of n-C_16_ hydroisomerization over the different catalysts at 340°C.

Products	Selectivity (%)				
	NiW/4Ni-SAPO-11	NiW/3Ni-SAPO-11	NiW/2Ni-SAPO-11	NiW/1Ni-SAPO-11	NiW/SAPO-11
i-C_16_ selectivity (%)	87.49	90.3	82.17	78.04	72.18
TOF (h^−1^)	27.71	30.81	29.80	28.44	27.70
2-MC_15_	16.52	16.97	15.23	14.24	13.57
3-MC_15_	25.67	26.46	24.43	23.52	22.65
4-MC_15_	11.31	12.27	11.91	11.28	10.17
5-MC_15_	12.61	12.01	11.05	10.63	9.38
6-MC_15_	4.49	5.95	5.07	4.46	4.54
2,4-DMC_14_	5.78	5.72	5.23	4.72	3.16
2,5-DMC_14_	4.31	3.91	3.45	3.34	2.92
2,6-DMC_14_	2.48	2.35	2.09	2.12	1.98
3,6-DMC_14_	0.93	1.37	0.97	0.93	1.13
3,7-DMC_14_	0.86	1.08	0.91	0.82	0.77
3-EC_14_	0.58	0.46	0.42	0.45	0.43
5-EC_14_	0.50	0.43	0.47	0.40	0.38
6-EC_14_	0.33	0.31	0.28	0.35	0.32
5,8-DEC_12_	0.31	0.25	0.15	0.24	0.28
Others	0.81	0.72	0.51	0.54	0.50

## Conclusion

SAPO-11 molecular sieves were modified with different Ni contents by the *in situ* modification method, and the Ni *in situ* modified SAPO-11 molecular sieves were used as the supports to prepare the NiW-supported catalysts for the hydroisomerization of n-hexadecane. The Ni *in situ* modified SAPO-11molecular sieves and the corresponding catalysts were characterized by a series of physicochemical characterizations. The results showed that Ni *in situ* modification increased the BET specific surface area, mesopore volume, and medium and strong Brønsted acid amounts of SAPO-11. For the corresponding NiW-supported catalysts, Ni *in situ* modification weakened the interaction between the active phase and support, which made the length of the so-called NiWS active phase slabs shorter, the number of stacking numbers higher, and the dispersion degree of the active phase higher. NiW/3Ni-SAPO-11 possessed the highest stacking number of the active phase and the highest dispersion degree of the active phase. The evaluation results of the hydroisomerization of n-hexadecane showed that due to the largest BET specific surface area, mesoporous volume, and medium and strong Brønsted acid amounts of 3Ni-SAPO-11; the appropriate interaction between the active phase and support; and the highest dispersion of the active phase of NiW/3Ni-SAPO-11, NiW/3Ni-SAPO-11 showed excellent catalytic activity and high selectivity for the hydroisomerization of n-hexadecane, and its maximum yield of isomeric hexadecane reached 71.18%. It is expected to provide new theoretical guidance for the design and preparation of high-efficiency SAPO-11 molecular sieve-based catalysts for the hydroisomerization of n-alkanes.

## Data Availability

The original contributions presented in the study are included in the article/Supplementary Material, and further inquiries can be directed to the corresponding author.
